# Impact of problematic mobile phone use among Nursing Students

**DOI:** 10.1192/j.eurpsy.2025.941

**Published:** 2025-08-26

**Authors:** A. M. Cybulska, K. Rachubińska, D. Schneider-Matyka, S. Mazurkiewicz, E. Grochans

**Affiliations:** 1 Department of Nursing, Pomeranian Medical University in Szczecin; 2 Independent Public Provincial Hospital in Szczecin, Szczecin, Poland

## Abstract

**Introduction:**

Technological development and the ever-expanding range of functions that a mobile phone offers to its users cause that more and more people overuse this device. The problem of smartphone addiction and nomophobia has become a significant social problem, especially among young mobile phone users. It has been observed that excessive use of smartphones can cause psychological problems, such as increased levels of stress, anxiety, depression, decreased cognitive function, and can also negatively affect students’ academic activities.

**Objectives:**

The primary aim of the study was to assess the extent of phone addiction among nursing students. Additionally, the researchers aimed to investigate the impact of various variables, including socio-demographic and psychological factors, on the severity of pathological phone use among nursing students.

**Methods:**

This survey based study was performed using the author questionnaire and standardized research tools: the Nomophobia Questionnaire (NMP-Q), the Mobile Phone Problem Use Scale for Adolescents (MPPUSA), the Athens Insomnia Scale (AIS), and the Depression, Anxiety, and Stress Scale version 21 (DASS-21).

**Results:**

The study involved 303 nursing students of the Pomeranian Medical University in Szczecin. A subjective feeling of addiction to a mobile phone was noted in 51.16% of respondents, and to the internet in 22.44%. In addition, 66.34% of the respondents had sleep problems. Some 38.28% of the nursing students did not show symptoms of depression, 38.61% had normal levels of anxiety, and 37.62% had normal levels of stress. Phone use was significantly more problematic among single people and those in an informal relationship, as well as among younger respondents. Analysis of the data revealed statistically significant positive correlations between problematic phone use according to the MPPUSA and the severity of depression, anxiety, stress, and insomnia according to the AIS.

**Table 1:** 

Variable	MPPUSA [score]		
	r		p
**NMP-Q**	No possibility of communication	0.531	**< 0.001**
	Loss of connectivity	0.6	**< 0.001**
	Being unable to get the news	0.682	**< 0.001**
	Inconvenience	0.64	**< 0.001**
**DASS-21**	Level of depression	0.324	**< 0.001**
	Level of anxiety	0.333	**< 0.001**
	Level of stress	0.404	**< 0.001**
**AIS**	0.317		**< 0.001**

**Conclusions:**

The vast majority of nursing students use a mobile phone correctly and do not exhibit symptoms of nomophobia. Age and marital status are the sociodemographic variables that have a statistically significant effect on the pathological pattern of smartphone use. There is no statistically significant relationship between mobile phone addiction and gender or place of residence. Phonoholism statistically significantly positively correlates with nomophobia, as well as the severity of depressive symptoms and insomnia. Moreover, the more pathological the smartphone use, the higher the levels of anxiety and stress experienced by nursing students.

**Disclosure of Interest:**

None Declared

